# Oral and maxillofacial surgery specialty awareness among medical undergraduates in North India: A cross-sectional study

**DOI:** 10.6026/973206300214629

**Published:** 2025-12-15

**Authors:** Ajit Kumar Vishwakarma, Manisha Upadhyay, Rajaram Yadav, Sanjeev Kumar Singh, Neeraj Kumar Dhiman, Naresh Kumar Sharma

**Affiliations:** 1 Department of Dentistry, Government Medical College, Azamgarh, Uttar Pradesh, India; 2 Department of Anatomy, Government Medical College, Azamgarh, Uttar Pradesh, India; 3 Department of Community Medicine, Government Medical College, Azamgarh, Uttar Pradesh, India; 4 Department of Dentistry, SMMH Government Medical College, Saharanpur, Uttar Pradesh, India; 5 Department of Oral and Maxillofacial Surgery, Faculty of Dental Sciences, IMS, Banaras Hindu University, Varanasi, India

**Keywords:** Dentistry, maxillofacial surgery, medical undergraduate

## Abstract

Oral and maxillofacial surgery though being a well-established specialty in India, still struggles for proper referrals from medical specialities. Majority of the referrals to this speciality turn up exclusively through dental practitioners while lacking the same from medical counterparts. The familiarity of medical undergraduates with the scope of Oral and maxillofacial surgery is vital to set the stage for appropriate patient referrals in future. Henceforth, a cross-sctional study was conducted among medical undergraduates in order to assess their understanding of this specialty by generating an electronic questionniare. A total of 301 participants from various medical institutions completed the questionnaire. Cronbach's alpha was employed to measure participants referral preferances for common maxillofacial surgery procedures. The study evaluated an alpha coefficient of 0.79 which suggests a justifiable reliability. Thus, this study shows the paramount importance of Oral and maxillofacial surgery awareness among medical undergraduates to pave the way for suitable referrals. Moreover, it recommends an obvious need for introduction and comprehensive exposure to this speciality as a part of medical curriculum.

## Background:

Oral and maxillofacial surgery (OMFS) being an internationally recognized surgical speciality is primarily a speciality of dentistry which requires only Bachelor of Dental Surgery (BDS) as a prerequisite in India and United states, while in United Kingdom, it requires dual qualification in medicine as well as dentistry to pursue OMFS speciality course [1, 2]. It is an area of specialization which is a mélange of medicine and dentistry offering wide diversity of management ranging from oral surgery, complex facial trauma, head and neck pathology together with subspecialization including head and neck oncology, reconstruction to craniomaxillofacial surgery [3]. Regardless of being well established speciality in India, it is battling for majority of befitting referrals together with other medical specialities like Otorhinolaryngology and Plastic surgery, as most of the referrals to the OMFS speciality crop up through dental practitioners while lacking the same from medical counterparts [4, 5-6]. This study aims to evaluate knowledge and exposure of OMFS speciality among medical undergraduates and their understanding about the role of oral and maxillofacial surgeons in management of head and neck conditions.

The whole idea of this study basically aims to analyse mindfulness of medical undergraduate students regarding the concept of OMFS as a branch bridging dentistry and medicine subsequently leading to appropriate patient referrals and optimal treatment outcomes since lack of clarity of boundaries between Otorhinolaryngology and even Plastic surgery often leads to potentially affect patient care [7, 8]. Therefore, it is of interest to gain Insight on Oral and Maxillofacial Surgery Specialty among Medical Undergraduates of North India.

## Methodology:

## Study design:

This cross sectional study resorted to a non-probabilistic cohort sampling approach. An electronic questionnaire was generated utilizing precursory literature and judgement of two expert Oral and Maxillofacial Surgeons for content validity [9]. The aim of the survey and the particulars about the questionnaire were explained to the participants prior to the survey itself. The potential partakers were well informed that their consent would be presumed by completion of the survey. Participants were invited to fulfill an online version of the questionnaire via email and social media from all possibly approachable medical institutions. Only medical undergraduates (Pursuing/Completed MBBS course, Non-Academic JRs) were included while academic JRs and students from dentistry background were excluded. To diminish potential bias during participant recruitment, we used voluntary and anonymous nature of participation and each recipient of the google form link was given one opportunity with an obvious choice of freedom regarding willingness to participate in the study and complete the online questionnaire at their own personal convenience.

## Variables:

The primary independent variable was classified as OMFS exposed or OMFS non- exposed among medical undergraduates. Covariables were Academic progression and Demographics of the participants. The main primary outcome varaibles were awareness of most ubiquitous OMFS procedures and referral patterns while secondary outcome variables were root of information of medical undergraduates concerning OMFS course as well as knowledge of eligibility required to obtain admission and further course duration of the speciality.

## Data collection:

Data from manifold cohort of medical undergraduates of copious medical college/institutions of India were stockpiled with recurrent promptings over a spectrum of three months spanning from December 2024 to February 2025 without any distinctive identifiables. To comprehend response bias and base referral knowledge, a randomized array of typical and atypical surgical conditions or clinical framework procured from OMFS curriculum were proferred accompanied with added assemblage of questions from independent medical specialities.

## Data analysis:

Subsequent to data collection phase, figures from electronically generated link were aggregated and interpreted using Stata 14.1 (@Copyright 1996-2025 StataCorp LLC) for windows. Descriptive statistical analysis is done for all the variables and Chi-square tests were applied to examine the association between categorical variables. Cronbach's alpha was calculated for five common surgical conditions or clinical scenarios (Facial injury / jaw fractures, Jaw correction surgery, Surgical pathology of face/ jaw/ oral cavity, 3rd molar surgery and Dental implant surgery) to check internal consistency of participants referral preferences for OMFS procedures. For all results, a p value of <0.05 was considered to be statistically significant.

## Results and Discussion:

An aggregate of 301 medical undergraduates from disparate medical institutions consummated the questionnaire. Roughly more males (60.5%) than females (39.5%) completed the questionnaire. Participants were disseminated athwart all academic stages of which 154 (51.16%) were from 1st professional, 65 (21.59%) from 2nd professional, 43 (14.29%) from final professional part-1, 21 (6.98%) from final professional part-2, 4 (1.33%) were intern, 4 (1.33%) were non-academic JRs/tutor/demonstrator and 10 (3.32%) were others. Table 1 (see PDF) presents the detailed demographic characteristic of the participants. Analysis of participants exposure to OMFS speciality during clinical posting/teaching/seminar/other methods reveals that out of 283 respondents to this question, only 43 (15.2%) were exposed while 240 (84.8%) were not exposed. In a wide range of surgical scenarios provided to the respondents, the relationship between OMFS exposed group, non-exposed group and referral pattern are depicted in (Table 2 - see PDF). Perception of common OMFS procedures in the form of referral pattern among OMFS exposed and non-exposed groups are depicted in Figure 1 (see PDF). Crobach's alpha was employed to estimate the internal consistency of the scale which measures participants referral preferances for common OMFS procedures. In our investigation, we evaluated an alpha coefficient of 0.79 which suggests a justifiable reliability. Linear trend for perception of common OMFS procedures in the form of referral pattern across academic status is depicted in Figure 2 (see PDF). We had provided a list of four qualifications to examine participants understanding of essential eligibility to get admission in OMFS speciality course. Out of 301 respondents, 47.5% were not acquainted with this qualification while only 24.6% selected Bachelor of Dental Surgery (BDS) as a prerequisite. We had also asked the participants to state the duration of OMFS speciality course and out of 288 respondents, only 22.9% correctly retorted to 3 year duration while 68.1% were not aware. Medical undergraduates apprehension regarding recurrent dental diseases, oral signs/symptoms of systemic diseases and maxillofacial infirmities that are life intimidating is not much crystalline. In the scenario of discernment of surgical procedures performed by oral and maxillofacial surgeons, most respondents realized that only facial injury, congenital facial deformity, jaw correction surgery, oral cancer, cleft lip palate and pathology of jaws were most frequently performed while face neck space infection, salivary gland diseases, third molar surgery, wisdom tooth, TMJ diseases, dental implant surgery were less frequently predictable as OMFS procedures. Nevertheless, oral and maxillofacial surgeons are customarily performing these procedures on an everyday basis. In point of the fact that OMFS is a part of dentistry and not medicine, so insight into latitude of OMFS speciality among medical undergraduates oftentimes lacks a clear demarcation between the ailments of oral cavity and those outside (face and neck), which at some point in the future eventually leads to inappropriate referrals [10].

This study entails a decent number of medical undergraduates from contemporary medical institutions of India and the evaluation lines up with antecedent publications revealing the fact that a huge predominance of medical undergraduates still are unveiled to OMFS speciality since overall the data revealed that only 43 respondents (15.2%) had a previous exposure to OMFS speciality while 240 (84.8%) had no idea of any such existing speciality [11]. The clinical exposure around academic progression plays a pivotal role in sculpting familiarity towards surgical conditions of the oral cavity, face and neck which is subsequently reflected in referral tendencies. Our digging up through this topic unveils a fluctuation in the discernment of routine OMFS procedures and therefore fortifies the role of OMFS exposure somehow or the other. While sweeping through frequent clinical scenarios individually, the study marked an undoubtable obligation for inclusion of the purpose of OMFS speciality among medical undergraduates. For example, facial injury being considered as a common OMFS procedure and hence marked appropriate in the context of referral to an oral and maxillofacial surgeon, out of 197 respondents, 68.3% were from non-exposed group while 76.4% belonged to the exposed group (p= 0.269; χ^2^= 1.219). Similarly, for jaw correction surgery, out of 144 respondents, 49.6% were from non-exposed group while 58.1% were the part of exposed group (p= 0.301; χ^2^= 1.068). Likewise, for surgical pathology of face/jaw/oral cavity, out of 122 respondents, 42.5% came from non-exposed group while 46.5% were belonging to the exposed group (p=0.625; χ^2^=0.239). Correspondingly, for 3rd molar surgery, out of 82 respondents, 28.8% were arising out of non-exposed group while 30.2% were coming from exposed group (p=0.844; χ^2^=0.039). This inclination is harmonious with quondam studies aiming at the climacteric role of OMFS exposure in whittling referral orientation [12, 13-14]. Nevertheless, Referral pattern for dental implant surgery was unpredictable and showed an unanticipated downturn since out of 100 respondents, 35.8% were from non-exposed group while 32.6% were belonging to the exposed group. This shift possibly might be related to the dispersion of implant placement among OMFS, Periodontists and Posthodontists as there is paramount role of these specialities in implant success [15, 16]. Strikingly, referral paradigm for more elaborate procedures (e.g. oral cancer, cleft lip and palate and salivary gland diseases) shows a modest elevation in preference among OMFS exposed group (Table 2 - see PDF). This situates very well with foregoing observations on the medical professionals insight on OMFS, and henceforth spotlights the exigency for acknowledgement to execute these changes in the field of OMFS [17, 18- 19]. Furthermore, there is noteworthy change in the segment of medics who believe that congenital facial deformity, face and neck space infection and TMJ diseases should preferentially be referred to an oral and maxillofacial surgeon. In a nutshell, greater proficiency, expertise and appeal to OMFS would consecutively lead to enhanced patient outcomes and eventually assist in eluding an emerging prospective workforce crisis sprouting as a result of unfamiliarity with this specialty [20].

## Conclusion:

The study showed that there is an evident necessity for introduction and panoramic exposure of oral and maxillofacial surgery specialty in the medical curriculum besides highlighting the contribution of oral and maxillofacial surgeons in head and neck diseases. It might be relevant to adjoin medical undergraduates teaching of OMFS with ENT (Otorhinolaryngology) in the form of an Assorted Head and Neck Capsule for superior understanding and better decisiveness in referral patterns for patient welfare. However, the study restrains to trace discrete changes for the whole of academic progression and emphasizes on fairly lesser number of medical institutions which freezes the universality of the conclusions.
